# On the importance of quality assurance (QA) for COMS eye plaque Silastic inserts: A guide to measurement methods, typical variations, and an example of how QA intercepted a manufacturing aberration

**DOI:** 10.1002/acm2.13325

**Published:** 2021-07-07

**Authors:** Courtney C. Oare, Christopher L. Deufel, Jordan McCauley Cutsinger, Tania De La Fuente Herman, Clara Ferreira

**Affiliations:** ^1^ Department of Radiation Oncology University of Minnesota Medical School Minneapolis MN 55455 USA; ^2^ Department of Radiation Oncology Mayo Clinic Rochester MN 55905 USA; ^3^ Department of Radiation Oncology University of Oklahoma Health Sciences Center Oklahoma City OK 73104 USA

**Keywords:** eye plaque, LDR brachytherapy, quality assurance, Silastic, uveal melanoma

## Abstract

**Purpose:**

Eye plaques are widely used for ocular melanoma and provide an effective alternative to enucleation with adequate tumor control. A COMS plaque utilizes a Silastic insert for precise positioning of the radioactive seeds with respect to the scleral surface of the eye; however, due to manufacturing variability, the insert may unintentionally increase or decrease the distance between the sources and tumor. The purpose of this work is to provide guidance in measuring and identifying outliers in Silastic inserts. The importance of regular quality assurance (QA) is illustrated in an experience where a systematic problem was detected and the manufacturer's 22‐mm mold was corrected.

**Methods:**

A detailed description of the molds and manufacturing process used to produce Silastic inserts is provided, including photographs of the process steps. The variability in Silastic insert production was evaluated by measuring the thickness of 124 Silastic inserts. An estimate of how the observed Silastic thickness discrepancies impact the dose to the tumor and critical eye structures was performed using homogeneous dose calculations. A standard QA protocol was developed to guide the clinical user.

**Results:**

Thickness of the measured Silastic inserts ranged from 1.22 to 2.67 mm, demonstrating variation from the 2.25 mm standard. Six of the 22‐mm inserts were outliers (Δthickness >3 standard deviations) and were excluded from the statistics. The outliers were investigated with the help of the manufacturer, who discovered that a systematic error was accidentally introduced into the 22‐mm mold.

**Conclusions:**

Due to manufacturing errors or variability, the Silastic inserts used in COMS eye plaques may be thicker or thinner than the design standard. Such variations may impact tumor control or increase the risk of normal tissue side effects. A standardized QA program is recommended to detect variations and communicate unusual findings to the manufacturer.

## 1 INTRODUCTION

Uveal melanoma is the most common type of intraocular tumor in adults with an incidence rate of 5.1 per million people.^1^ In the United States, roughly 1400 cases are diagnosed annually.^2^ Most uveal melanomas arise from the choroid (90%) but also can occur in the iris or ciliary body.^3^ Without treatment, the eye becomes painful, visually impaired, and metastatic disease is likely. Brachytherapy is the mainstay treatment method to achieve tumor control and prevent local recurrence. The Collaborative Ocular Melanoma Study (COMS) group was formed in 1985 to evaluate outcomes of episcleral plaque brachytherapy. The study confirmed that, for medium‐sized tumors, enucleation of the eye and brachytherapy have comparable survival outcomes, whereas brachytherapy may be more cosmetically appealing than enucleation.^4^, ^5^, ^6^ Alternative treatment methods include proton beam therapy or Gamma Knife stereotactic radiotherapy.^7^ For medium‐sized tumors, plaques are typically chosen due to availability, cost, and historical outcomes.^8^

**FIGURE 1:**
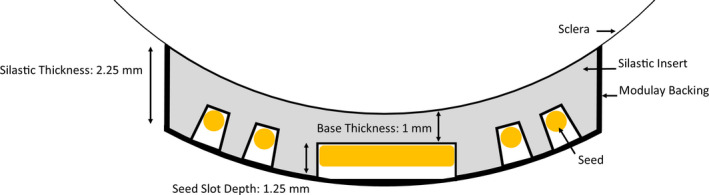
Nominal design of a standard COMS plaque and Silastic insert. A change in base thickness would shift the radioactive seeds parallel away from the sclera (too thick) or toward the sclera (too thin)

In addition to proving the effectiveness of plaque therapy, COMS developed a standardized eye plaque and radioactive seed loading. Manufactured by Trachsel Dental Studio (Rochester, MN), a standard COMS plaque consists of a gold‐alloy backing with a collimating lip. Inside this backing rests a Silastic insert, which holds radioactive seeds in reproducible positions. The Silastic insert is designed to have a 1‐mm‐thick “base layer” to create a small separation between seeds and the scleral surface of the eye.^6^ The Silastic mold also has 1.25‐mm‐tall spacers to provide the “slots” for seed placement, indicating that the entire plaque should have a nominal thickness of 2.25 mm.^9^ Figure 1 illustrates a COMS plaque and its relevant components according to the COMS Manual of Procedures.^9^ I‐125 and Pd‐103 are commonly used sources for COMS plaques, both gamma emitters with average energies of 28 and 21 keV, respectively.^10^ I‐125 has a half‐life of 59.4 days, and Pd‐103 17 days.

**FIGURE 2:**
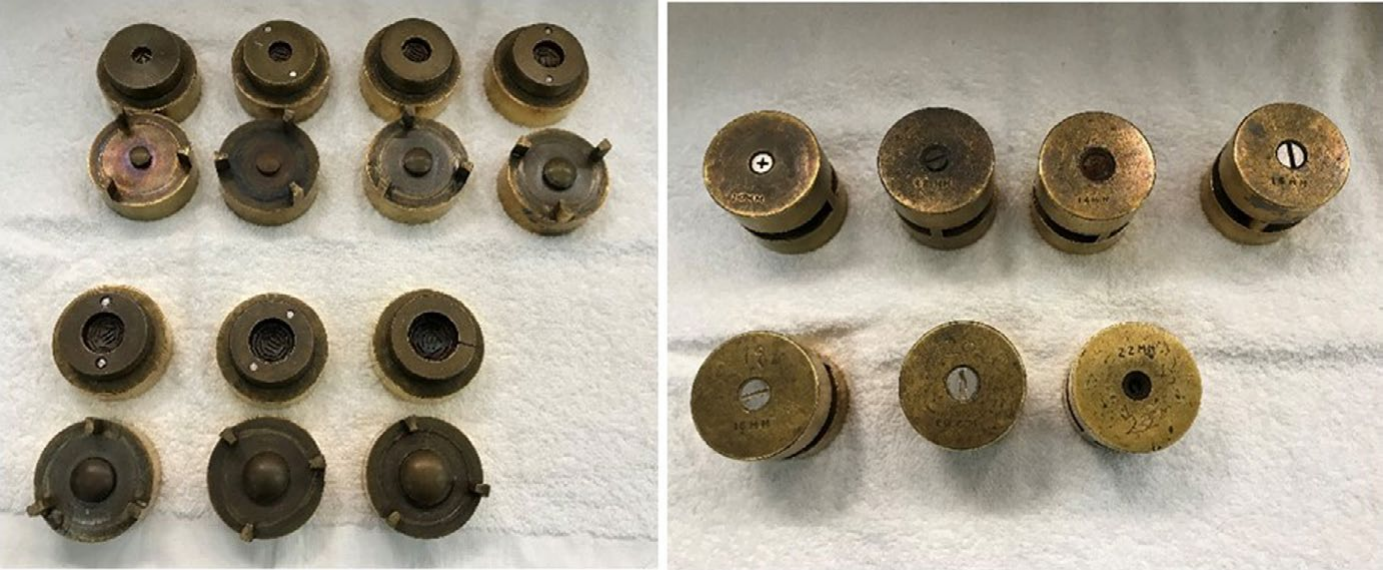
The seven brass molds that have been used to produce all COMS Silastic inserts to date are shown. *Left*: Open molds, *Right*: Closed molds

**FIGURE 3:**
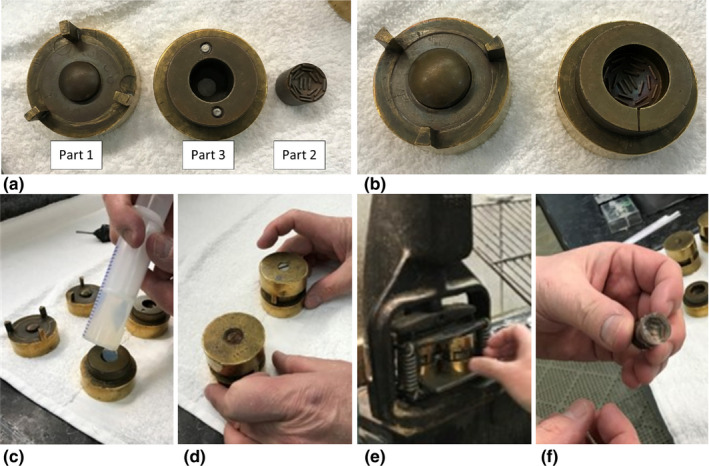
The Silastic mold is comprised of three parts, each of which was machined from brass. Part 1 contains a convex surface with radius of curvature equal to 12.3 mm (pictured far left in Figure 3a). Part 2 is a cylinder whose top surface is concave with radius of curvature equal to 14.3 mm (pictured far right in Figure 3a). The concave surface contains metal shims that protrude 1.25 mm from the surface to create seed slots. Part 3 is a holder for Part 2 and precisely creates a 2‐mm gap between the convex and concave surfaces of Part 1 and Part 2. To create a Silastic insert, Part 2 is placed inside of Part 3 (Figure 3b), and raw Silastic material is poured into the concave surface of the Part 2/3 assembly (Figure 3c). Part 1 is placed atop the Part 2/3 assembly and clamped to maintain pressure (Figure 3d), whereas the material cures in a warm water bath (Figure 3e). After curing has completed, the parts are disassembled, and the Silastic is removed (Figure 3f)

All Silastic inserts manufactured for COMS plaques originate from a unique set of seven molds that were manufactured by Mayo Clinic in the late 1980s and early 1990s and are currently owned by Trachsel Dental. The 10‐ and 22‐mm‐diameter molds appear to have been created later than the others as the need for smaller/large tumor coverage became apparent. A single mold exists for each of the seven standard COMS sizes: 10, 12, 14, 16, 18, 20, and 22 mm (Figure 2). Figure 3 highlights the Silastic molding process.

It is expected that there will be systematic and random variation in the Silastic manufacturing process. Systematic errors may include the seed slot depth as defined by the metal “seed” shims, which are press‐fit into Part 2. This error would change the seed slot depth. Furthermore, a discrepancy in the gap between the Part 2/3 assembly and Part 1 or the radii of curvature of the components can produce systematic errors that would alter the base thickness (distance between the bottom of the seed slot wells and the outer edge). Random discrepancies may arise from how much material is poured into the mold, the pressure used to hold the components together, and the behavior of the material during the curing process (e.g., shrinkage).

The American Association of Physicists in Medicine (AAPM) Task Group 56 recommends that brachytherapy applicator dimensions, integrity, and dosimetric properties are evaluated initially and annually.^11^ AAPM recognizes that applicator dimensions should be within 0.5 mm of manufacturer specifications. In addition, regarding eye plaques, AAPM Task Group 221 recommends that the positions (excluding the depth) of the seed slots can be measured with a flatbed scanner, achieving agreement with manufacturer design within 0.2 mm.^12^ A flatbed scanner can provide seed positions; however, it should be noted that compression inside the scanner may occur. Currently, no consensus exists to appropriately measure Silastic insert thickness or seed slot depth.

The purpose of this work is to evaluate how the manufacturing and molding process may lead to variation in the thickness of COMS Silastic inserts. To that end, a simple method for measuring the thickness of Silastic inserts is proposed, and inter‐ and intra‐observer variability was quantified. The approach was used to measure a large quantity of Silastic inserts of all sizes to better understand the systematic and random variations. The importance of such QA was illustrated with an experience in which a 22‐mm COMS Silastic mold was modified by the manufacturer to meet the needs of another institution, creating a systematic change in several Silastics that were produced. The issue was detected during regular QA, and the clinical user and manufacturer worked together to correct the problem. This work proposes adding Silastic thickness measurements to a standardized QA program and recommends guidance if thickness discrepancies are observed.

## 2 MATERIALS AND METHODS

### 2.1 Physical thickness measurements

**FIGURE 4:**
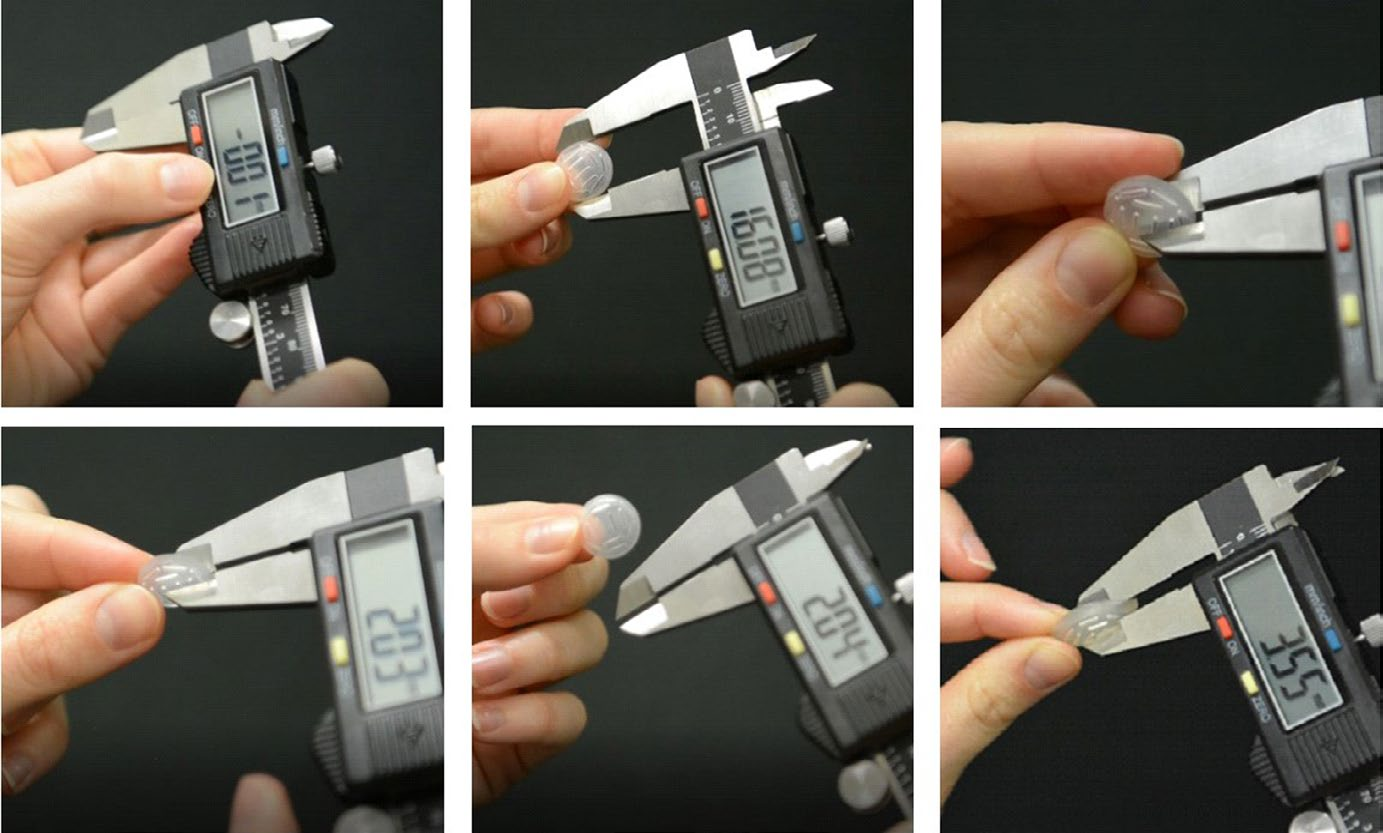
Demonstration of Silastic thickness measurement with a digital caliper. *Top left to right*: The caliper is zeroed, the Silastic diameter is confirmed to be 16 mm, and the Silastic is inserted into the caliper's jaws. *Bottom left to right*: The Modulay thickness is not distorted by the caliper jaws, and a measurement is taken. The Modulay should be able to freely move out of the gap created without changing the measured thickness; the final measurement is 2.04 mm. The bottom right image displays measurement of a large, 3.55‐mm‐thick insert

One hundred twenty‐four Silastic inserts, diameters ranging from 10 to 22 mm, were measured utilizing digital calipers from one of two institutions: Site A or Site B. The base thickness (e.g., nominal thickness =1 mm) was obtained by measuring the overall thickness of the Silastic material and subtracting the depth of the seed slot. Site A used an American National Standards Institute (ANSI) digital caliper and a depth gauge, and Site B utilized a digital caliper.^8^ Both methods provided submillimeter precision and were readily available at each clinic. Measurements were performed gently as to not compress the Silastic material. Figure 4 describes measurement using a Silastic insert and may guide the clinical user.

The seed slot depth was verified by cutting a small number of used Silastic inserts in half and measuring the total thickness and base thickness with a digital caliper. The difference between the total and base thickness is the seed slot depth. Measurements were performed at both central and peripheral slot points. The mean, maximum, and minimum slot depth are recorded for each plaque diameter. Measurements were performed for 12‐ to 20‐mm plaque diameters as used Silastic inserts for 10‐ and 22‐mm‐diameter plaques were not available at the time of measurement.

### 2.2 Intra‐observer, inter‐observer, and inter‐instrument variation

The intra‐ and inter‐observer variability in the measurement of a Silastic insert was evaluated for each Silastic insert size. A total of 63 measurements were performed. Two different users performed three repeated measurements of each insert using the same digital caliper. Additionally, one of the users performed three measurements of each insert using a different digital caliper. The variations were calculated according to Equations (1)–(6), where *M*(user, instrument, Silastic size, trial) represents one measurement by a given user, instrument, Silastic size, and trial and *M*_trial_(user, instrument, Silastic size, trial) represents the average over three trials for a specific user, instrument, and Silastic size.

(1) $$\begin{array}{cc} & \text{Average}\;\text{intra - observer variability}\\ & \;=\frac{1}{21}\sum \left|M\left(user,\;instrument,Silastic\;size,\;trial\right)‐\bar{M_{\mathrm{trial}}}\left(user,\;instrument,Silastic\;size\right)\right| \end{array}$$

(2) $$\begin{array}{cc} & \text{Maximum}\;\text{intra - observer variability}\\ & \;=Max\left|M\left(user,\;instrument,Silastic\;size,\;trial\right)‐\bar{M_{\mathrm{trial}}}\left(user,\;instrument,Silastic\;size\right)\right| \end{array}$$

(3) $$\begin{array}{cc} & \text{Average}\;\text{inter - observer variability}\\ & \;=\frac{1}{7}\sum \left|\bar{M_{\mathrm{trial}}}\left(user\;1,\;instrument\;1,Silastic\;size\right)‐\bar{M_{\mathrm{trial}}}\left(user\;2,\;instrument\;1,Silastic\;size\right)\right| \end{array}$$

(4) $$\begin{array}{cc} & \text{Maximum intra - observer variability}\\ & \;=Max\left|\bar{M_{\mathrm{trial}}}\left(user\;1,instrument\;1,\;Silastic\;size\right)‐\bar{M_{\mathrm{trial}}}\left(user\;2,\;instrument\;1,\;Silastic\;size\right)\right| \end{array}$$

(5) $$\begin{array}{cc} & \text{Average}\;\text{inter - instrument variability}\\ & \;=\frac{1}{7}\sum \left|\bar{M_{\mathrm{trial}}}\left(user\;1,\;instrument\;1,\;Silastic\;size\right)‐\bar{M_{\mathrm{trial}}}\left(user\;1,\;instrument\;2,\;Silastic\;size\right)\right| \end{array}$$

(6) $$\begin{array}{cc} & \text{Maximum}\;\text{inter - instrument variability}\\ & \;=Max\left|\bar{M_{\mathrm{trial}}}\left(user\;1,\;instrument\;1,\;Silastic\;size\right)‐\bar{M_{\mathrm{trial}}}\left(user\;1,\;instrument\;2,\;Silastic\;size\right)\right| \end{array}$$

### 2.3 Dosimetric calculations

The dosimetric impact of variations in the base thickness of Silastic inserts was evaluated using I‐125 seeds (IsoAid Advantage, Model IAI‐125) with a commercial treatment planning system (TPS), Eclipse BrachyVision (Version 11.0.47, Varian Medical Systems, Palos Altos, CA) using the AAPM Task Group 43 updated formalism.^13^ The TPS calculated dose to water, not correcting for heterogeneities. In a virtual water phantom, 12‐, 14‐, 16‐, 18‐, 20‐, and 22‐mm COMS plaques were created, in which the seeds are placed according to AAPM Task Group 129.^14^ A prescription dose of 8500 cGy was prescribed to the tumor apex, as recommended by the American Brachytherapy Society.^15^

For the dosimetric modeling, an individual patient was selected with the tumor located posterior in the eye, having a base of 18 mm in diameter, located 2 mm from the optic disc. The eye coordinate system places the origin at the eye center, the x‐axis extends from lens to fovea, and the y‐axis extends right to left.^16^ Normal tissue structures and their corresponding reference point locations in the eye coordinate system include the inner sclera (−2.7, 10.7, 0), optic disc (−10.9, 1.3, 0), fovea (−11, 0, 0), lens center (7.5, 0, 0), opposite retina (0, 0, 22), and the tumor apex (0, −1, 0). Only one patient eye was used in our analysis to demonstrate the dosimetric impact of Silastic thickness changes.

Estimates for the dosimetric impact of Silastic thickness deviations were estimated by increasing or decreasing the distance between the radioactive seeds and the reference points. The methodology for modeling the change in seed positions depends upon the anticipated physical cause of Silastic deviations. For example, if the anticipated cause is that Part 2 is sitting too low or high inside of Part 3, then all radioactive seeds inside of the insert would be expected to shift along a direction parallel to the plaque central axis. This can be visualized as an increased base thickness (>1 mm nominal thickness) compared with that displayed in Figure 1.

To simulate the shift of radioactive seeds parallel to the plaque in the TPS, the critical structure and prescription points were displaced relative to the seed collection in 0.5 mm increments: −0.5, 0.0, +0.5, +1.0, +1.5, and +2.0. An insert that was 2 mm larger than the nominal thickness was represented by +2.0 mm, whereas a thinner Silastic insert was represented by −0.5 mm. Dose changes to the tumor apex were calculated for a prescription depth equal to 5 mm from the inner sclera, plaque diameters (12–22 mm), and the various Silastic thickness deviations. It should be noted that at the time of calculations, the 10‐mm plaque was not available in the TPS where measurements were performed.

Additionally, changes to the normal tissue structures were considered. The change in dose to the inner sclera, optic disc, fovea, lens, and opposite retina were calculated using a 22‐mm plaque for prescription depths of 5, 7.5, and 10 mm. The 22‐mm plaque was specifically chosen because it represents the plaque which was associated with a manufacturing error. Alternatively, the silastic thickness could be incorrect because the radius of curvature of Part 2 is incorrect, and all radioactive seeds inside of the insert would be expected to shift along a radial direction, $\hat{R}$, from the center of the eye. Such effects have been previously quantified by Johnson et al.^4^

## 3 RESULTS

### 3.1 Physical thickness measurements

**FIGURE 5:**
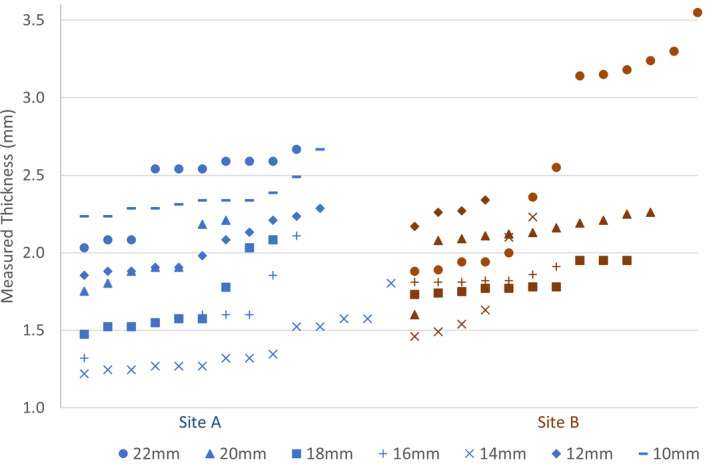
Physical measurements of Silastic inserts are shown. Each data point represents a unique Silastic insert, measured by Institution A (blue label) or Institution B (red label). The six largest 22‐mm‐diameter Silastic inserts from Site B were outliers and due to a manufacturing mistake

**FIGURE 6:**
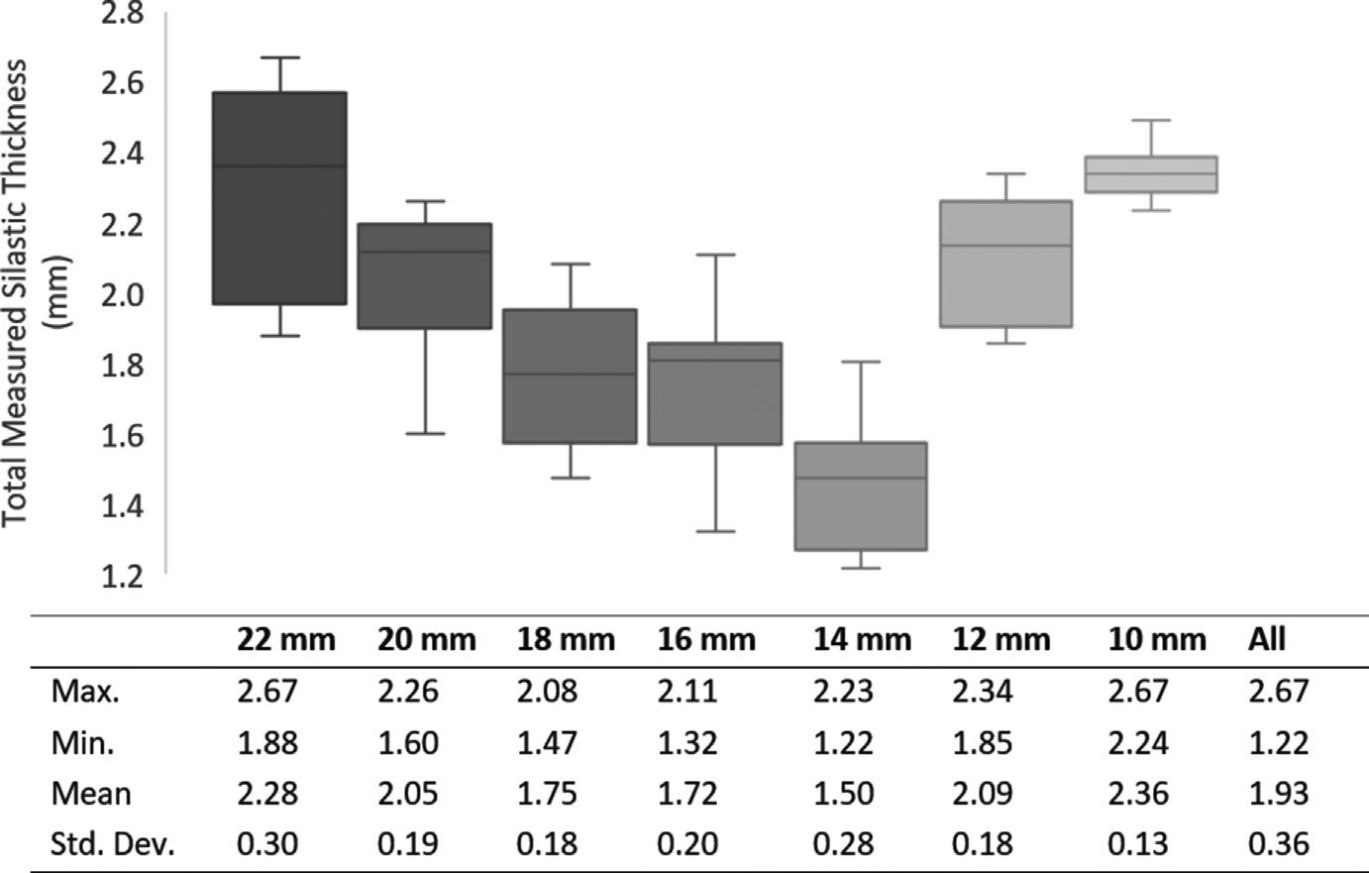
Box and whiskers plot of Silastic insert thicknesses distribution. Each box represents the 25% and 75% quartiles, the whiskers represent the minimum and maximum, and the bar represents the mean. The mean of inserts measured was 1.93 mm, and the standard deviation was 0.36 mm. Six outlier 22‐mm inserts (not included in this plot and table) ranged from 3.14 to 3.55 mm thick

Figure 5 displays the Silastic total thickness measurements from each institution (which were indexed from smallest to largest measurements) for all plaque sizes. The statistics for each plaque size are summarized in the box and whisker plot shown in Figure 6, excluding outlier measurements. The mean thickness of all inserts, excluding outliers, was 1.93 mm, with a standard deviation of 0.36. Six of the 22‐mm inserts were outliers (Δthickness >3 standard deviations from the mean) that were excluded from the figure. The thickness of these inserts ranged from 3.14 to 3.55 mm. Please refer to the discussion section for a description of our investigation into the cause of the deviations and follow‐up actions.

Table 1 highlights the mean, maximum, and minimum slot depths measured for each plaque diameter. Compared with the nominal slot depth of 1.25 mm, the 25 used Silastic inserts measured a mean slot depth of 1.18 mm, ranging from 1.05 to 1.29 mm. The base depth measured on average 0.07 mm greater at the center than the periphery of the Silastic.

**TABLE 1:** Measured slot depths for each Silastic diameter

Plaque diameter	Count	Mean slot depth (mm)	Minimum slot depth (mm)	Maximum slot depth (mm)
12 mm	2	1.13	1.05	1.21
14 mm	4	1.25	1.20	1.29
16 mm	3	1.17	1.12	1.22
18 mm	3	1.16	1.05	1.25
20 mm	13	1.19	1.14	1.23
All	25	1.18	1.05	1.29

### 3.2 Intra‐observer, inter‐observer, and inter‐instrument variation

**FIGURE 7:**
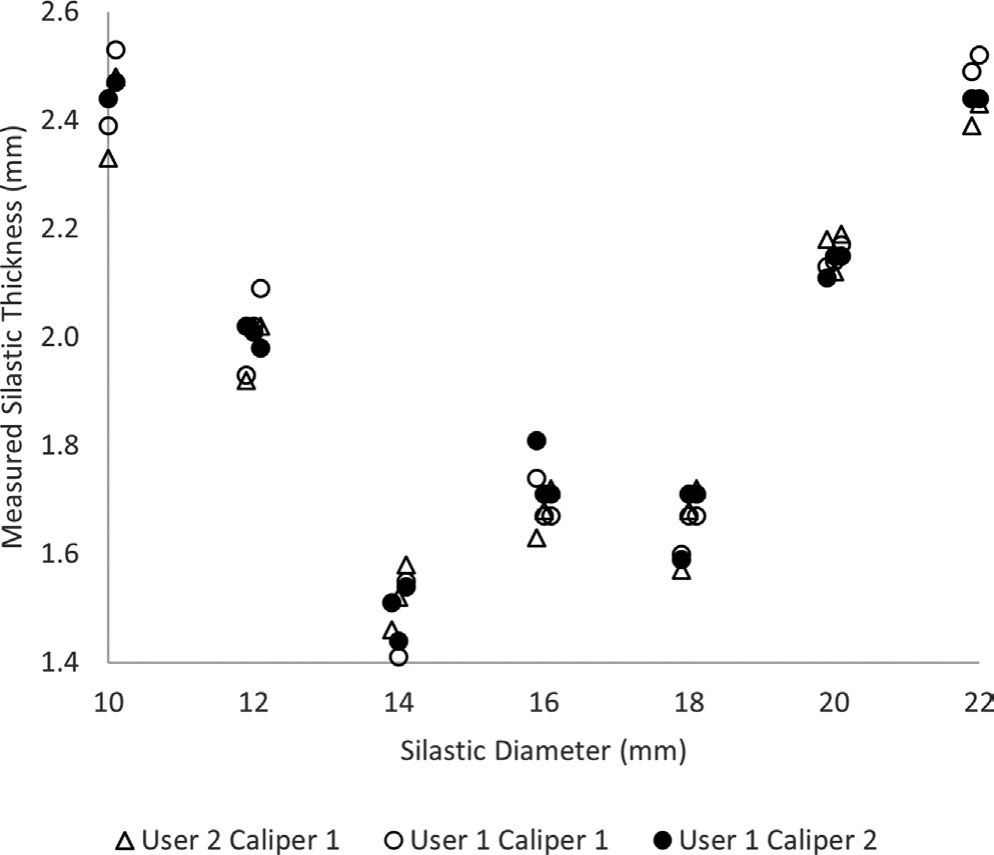
Variability of measurement for one Silastic insert of each diameter with a digital caliper is graphically shown for two users and two instruments

Figure 7 summarizes the intra‐observer, inter‐observer, and inter‐instrument results. The mean (maximum) intra‐observer variability from three repeated measurements was 0.03 (0.10) mm. The mean (maximum) inter‐observer variability from two users employing the same caliper was 0.04 (0.10) mm. The mean (maximum) inter‐instrument variability, one user performing measurements with two calipers, was 0.04 (0.11) mm. Overall, two users performing a single measurement with different calipers had a maximum total variation <0.23 mm.

### 3.3 Dosimetric calculations

The dosimetric impact of Silastic insert thickness deviations was evaluated for the situation where the deviation in Silastic base thickness is due to systematic movement of the radioactive seeds along a direction parallel to the plaque central axis. Table 2 summarizes how the dose to the tumor apex changed by −14% to −20% (thicker Silastic) and +16% to +25% (thinner Silastic) per millimeter of Silastic base thickness deviation. The percent dose changes were greater for smaller plaques and for deviations that corresponded to thinner Silastic inserts. The results are similar to work done by Johnson et al., who reported 16.5% and 20.3% deviation per millimeter for 22‐ and 16‐mm plaques, respectively, at a 5‐mm prescription depth.^4^

**TABLE 2:** Change in the dose to the tumor apex, %, for various ΔSilastic thickness

Plaque diameter (mm)	−0.5 mm	0 mm	+0.5 mm	+1 mm	+1.5 mm	+2 mm
22	8.2%	0.0%	−7.4%	−14.2%	−20.5%	−26.3%
20	8.7%	0.0%	−7.8%	−15.0%	−21.5%	−27.5%
18	9.5%	0.0%	−8.4%	−16.1%	−22.9%	−29.3%
16	10.5%	0.0%	−9.2%	−17.5%	−24.8%	−31.4%
14	11.5%	0.0%	−10.0%	−18.8%	−26.6%	−33.3%
12	12.7%	0.0%	−10.8%	−20.0	−28.1%	−35.0%

Note

Impact of Silastic insert thickness variation on the dose to the tumor apex, reported as % dose deviation. The tumor apex was located at a depth of 5 mm from the inner sclera. The thickness variation was modeled as a systematic shift of all radioactive seeds in a direction parallel to the plaque's central axis (+ = thicker, − = thinner).

Table 3 provides the change in the dose to the normal tissue structures for a 22‐mm‐diameter plaque, as a function Silastic thickness variation. For the tumor in the previously described location, with a 5‐mm prescription depth, the dose changed by −6%/mm (fovea), −8% (optic disc), and −64%/mm (inner sclera).

**TABLE 3:** Change in, %, for Silastic thickness variations

ΔSilastic (mm)	−0.5	0	+0.5	+1
Apex	8.2%	0.0%	−7.4%	−14.2%
Inner sclera	15.7%	0.0%	−36.5%	−63.9%
Optic disc	8.5%	0.0%	−4.1%	−7.7%
Fovea	7.5%	0.0%	−2.9%	−5.5%
Lens center	6.7%	0.0%	−1.7%	−3.3%
Opp. Retina	5.4%	0.0%	−0.5%	−1.1%

Note

The % change in the dose to the tumor apex and normal tissue structures as a function of Silastic insert thickness and prescription depth (+ = thicker, − = thinner). Doses are reported for a specific patient utilizing a 22 mm diameter located posterior in the eye and a 5‐mm prescription depth. The thickness variation was modeled as a systematic shift of all radioactive seeds in a direction parallel to the plaque's central axis.

## 4 DISCUSSION

### 4.1 Clinical impact

The Silastic insert fabrication process is manual, time consuming, and subject to systematic and random variations. Silastic inserts are fabricated one at a time because there is only one mold for each size. The systematic variations are represented by the difference in the mean thickness of the six plaque sizes and can be attributed to construction differences in each brass mold. In this study, the systematic variations were observed to range from −0.5 (14‐mm plaque) to +0.36 mm (10‐mm plaque). Random variations also occur during the production of each Silastic insert, possibly due to the amount of Silastic that is injected, how the mold pieces are seated together, and/or variations in environmental conditions. The random variations were represented by standard deviations that ranged from 0.13 to 0.30 mm.

**FIGURE 8:**
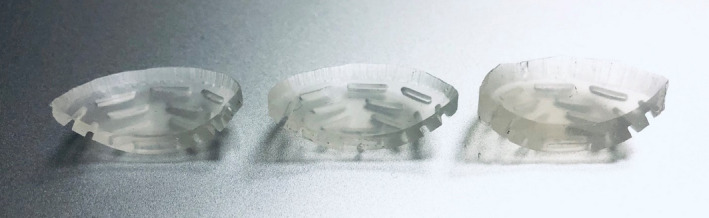
Images of three 22‐mm Silastic inserts, whose total thickness measured (from left to right) 1.90, 2.04, and 3.55 mm. The Silastic base thickness increases, whereas the seed slot depth remains unchanged

This work also underscores how an accidental error can be introduced into a Silastic mold. The inspiration for this manuscript was a large deviation that was observed in a new batch of 22‐mm Silastic inserts that arrived at the clinic. The inserts were noticeably of unusual thickness. The lower right image of Figure 4 shows the measurement of an unusually thick 22‐mm plaque. In this case, the Silastic insert measured 3.55 mm (above the suggested range, 2.25 ± 0.5 mm) and was *not* to be used with patients. Figure 8 provides a cross section of the insert alongside two “normal” Silastic inserts. The cause of this discrepancy was investigated on‐site with the manufacturer of the Silastic inserts. The manufacturer described how the position of Part 2 with respect to Part 3 in the 22‐mm Silastic mold had been recently lowered by ~1 mm at the request of a customer to create a thicker Silastic, which is possible by adjusting the gap between the components of the seed mold. The customer had a 22‐mm Modulay with an unusually high lip and desired a Silastic that better fit their Modulay. Presumably, the customer wanted deeper seed slots, and not a thicker Silastic base, but they were unaware of how changes to the mold would impact the base thickness. One of our coauthors assisted the vendor in restoring the original mold gap and discussed how the vendor could perform quality control before mailing Silastic inserts to other users. Additionally, the vendor helped to notify any potential recipients of the unusually thick Silastic inserts and provide them with replacements. Nevertheless, this experience highlights the importance of QA at the end user level.

The importance of submillimeter accuracy in the fabrication of Silastic inserts is displayed in Tables 2 and 3. The results illustrate that small changes in the Silastic thickness have a substantial impact on target dose and organs at risk. For Silastic inserts that are too thick, underdosing the target can lead to inadequate cell kill and potentially local recurrence or increased risk of metastasis. When the Silastic insert is thinner than expected, the dose to the sclera increases and may cause negative side effects such as scleral necrosis and additional eye pain. Other key structures impacted by thin Silastic inserts include the optic disc and the lens. Optic neuropathy and cataract formation have high incidence with increasing radiation doses.^17^, ^18^, ^19^, ^20^ For example, using a 1.5‐mm Silastic thickness (0.5 mm too thin) will result in dose to the optic disc that is 4% higher and dose to the inner sclera that is 16% higher. The inner scleral dose is of particular concern as high dose (>400 Gy) may result in scleral necrosis.^21^, ^22^ There is fortunately an upper bound on the decrease of the Silastic thickness because deviations greater than 1.0 mm will result in seed “holes” instead of seed slots.

Deviations in distance errors, including the Silastic thickness, could also lead to a reportable medical event. A reportable event occurs when the total dose differs from the prescribed dose by 20% or more according to 10 CFR 35.304. For instance, according to the work presented, the dose to the prescription point for a 22‐mm plaque prescribed to a depth of 5 mm from the inner sclera will change by 14.2% per mm of additional Silastic thickness when accounting for geometry alone, falling close to the regulatory limit. Such regulatory requirements should be kept in mind for QA programs and tolerance specifications.

### 4.2 Clinical recommendations

Because variability in Silastic insert thickness can occur due to random or systematic manufacturing errors, it is important that the physicist or dosimetrist verify the slot depth and base thickness for each insert that is used for patient treatment. The authors recommend that clinics avoid the use of Silastic inserts that are greater than 0.5 mm different from the overall thickness of 2.25 mm.

The measurement of the total Silastic thicknesses may be performed with a digital caliper or depth gauge.^8^ The device should report significant figures to 0.01 mm to obtain adequate precision. The caliper's accuracy can be double‐checked by measuring known dimensions of an available object, such as a solid water piece. Figure 4 demonstrates the use of a digital caliper to measure the total thickness of the Silastic insert. The curved Silastic is flattened between the jaws of the caliper. The measurement device should not overly compress the Silastic, which could underestimate thickness. Multiple measurements of the numerous parts of the Silastic are encouraged, because the Silastic may have variable thickness across its surface due to manufacturing differences in the radius of curvature of the convex (Part 1) and concave (Part 2) portions of the Silastic mold.

**FIGURE 9:**
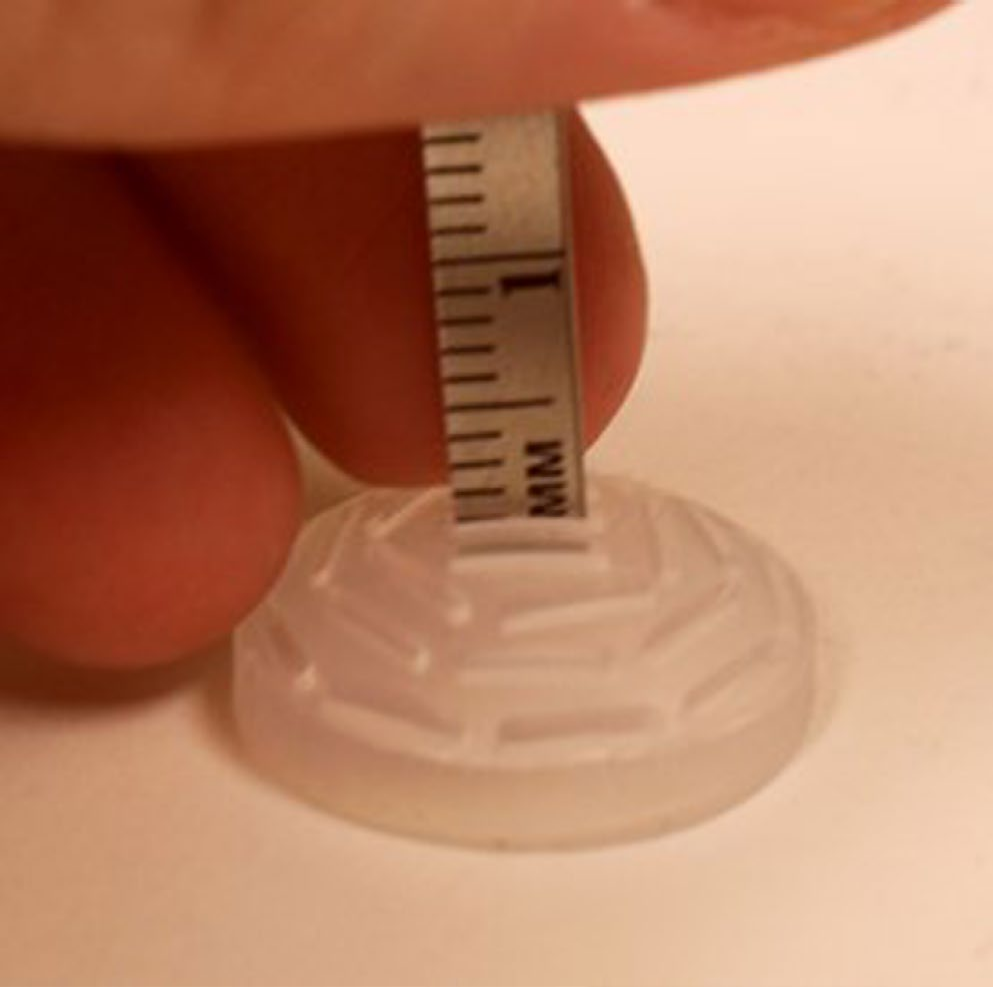
An inexpensive slot depth measurement tool was made by cutting a metal ruler lengthwise to fit in the narrow seed slot depth. The tool was used to ensure slot depths were 1.25 ± 0.5 mm by verifying that the slot is nearly 1.25 mm

The slot depth may be measured using a Vernier depth bar or depth gauge or with a modified ruler to ensure 1.25 mm depth at the plaque center. For routine clinical use, the authors recommend creating a thin tool to measure depth without cutting and sacrificing the insert. For example. see Figure 9, where a metal ruler was cut lengthwise to fit in the seed slot and is used to ensure that the seed slot depth at center is approximately 1.25 ± 0.5 mm. It is possible to create a “pass” or “fail” test using a simple measurement tool as described.

Based on the observed variation in Silastic thickness, clinics utilizing eye plaque brachytherapy are encouraged to establish QA programs for current and new Silastic inserts, noting discrepancies from the nominal thickness. Measurements should be repeated for precision, and equipment used should be cross‐checked for accuracy. Although there is no current recommendation by the ABS or AAPM on Silastic insert acceptance, we recommend that if the Silastic thickness varies by more than 0.5 mm from the 2.25 mm nominal thickness, the insert should not be utilized for patient treatment and the manufacturer should be contacted. Processes should be implemented for ongoing vigilance to limit future discrepancies.

### 4.3 Study limitations

The changes to the target and organ‐at‐risk doses calculated in the TPS underestimate the actual differences because they are not corrected for plaque inhomogeneities, including the attenuation of the Silastic insert. The electron density of Silastic (Z_eff_ =11) is larger than water (Z_eff_ =7.4).^14^ For low‐energy I‐125 photons, with a mean energy of 28 keV, the photon interactions in the medium are primarily due to the photoelectric effect, which is dependent on Z.^3^ With a higher Z, Silastic is more attenuating than water, and the actual dose changes are expected to be greater than what is reported here with increasing Silastic thickness. Astrahan et al. concluded that at 1 cm from the source, up to 10% dose reduction exists due to attenuation from 1 mm of Silastic material compared with no attenuation correction.^23^ Deufel et al. reported that 1 mm of Silastic base produces a correction factor equal to between 8% and 18%, depending upon the prescription depth.^4^, ^24^ The doses reported in this work ignore the attenuation effects of Silastic material. However, in future work, Monte Carlo studies could better account for Silastic attenuation to provide accurate dose from low‐energy gamma rays.

An additional limitation of this study was that only point doses reported for the normal tissue structures and only one plaque position was modeled. Volumetric doses may have a different response to Silastic thickness variations. The location of normal tissues with respect to the tumor will also be different for every patient, and therefore, the doses references herein are used to illustrate the clinical relevance of Silastic QA.

**FIGURE 10:**
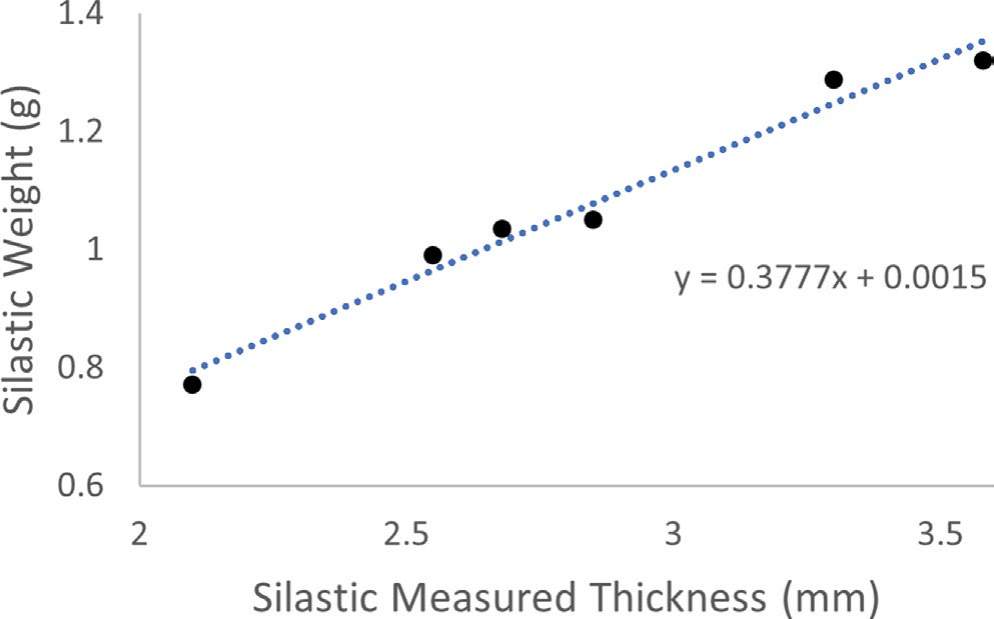
A direct relationship is seen among 22‐mm Silastic inserts with measured thickness and increasing weight. The Silastic weights had a linear trend with measured thickness with an *R* ^2^ = 0.98

Furthermore, Silastic material is nonrigid and curved and can be challenging to measure. Despite excellent precision of digital calipers, variation exists between instruments and measurements. For this reason, weight measurements are a proposed QA method to circumvent uncertainties of caliper measurements. If Silastic thickness increases, it is hypothesized that the weight will increase for the Silastic diameter. Preliminary data shows measured weight increases with measured Silastic thickness. Figure 10 shows the correlation between measured thickness and weight for six 22‐mm Silastics, including the large outliers. A linear fit is displayed among all 22‐mm Silastics (including outliers) with increasing weight and measured thicknesses, achieving an *R^2^* value of 0.98. From the above data, it can be concluded that an accurate 22‐mm thick insert should weigh about 0.75 ± 0.2 g. Future work is needed to better quantify the standard Silastic weight for each diameter based on a larger data set.

## 5 CONCLUSION

The manufacturing process of Silastic inserts for COMS eye plaques is susceptible to random and systematic errors that can produce deviations from the nominal thickness of 2.25 mm. Silastic inserts that are too thick or too thin can lead to underdosage of the target or overdosage of normal tissues. A Silastic QA program is encouraged for all clinics utilizing COMS eye plaques for the benefit of high‐quality patient treatment and outcomes. Silastic inserts that deviate more than 0.5 mm from the nominal thickness should be avoided for patient use. As demonstrated, communication between the clinic and manufacturer is essential to understand cause of discrepancies and avert future complications in brachytherapy and radiation oncology applications.

## 6 AUTHOR CONTRIBUTION STATEMENT

CLD and JMC originally identified the problem of unusually thick Silastic inserts, contacted the manufacturer, and led a discussion to prevent and correct the problem. All authors contributed to measurement data using their institution's inventory. Variability was quantified by CCO and CLD. Pictures were taken by CF, CLD, and CCO. CCO summarized the collective data and generated tables and figures. TDH suggested using weight measurements to identify Silastic inserts more accurately flag Silastic inserts that deviate from the standard thickness. All authors have reviewed and accepted the final version of this manuscript.

## CONFLICT OF INTEREST

No conflicts of interest.

## Data Availability

The data that support the findings of this study are available from the corresponding author upon reasonable request.
